# Relationship Between Quantitative MRI and Radiological, Histological, and Biochemical Measures of Intervertebral Disc Health in Client‐Owned, Nonchondrodystrophic‐Breed Dogs

**DOI:** 10.1002/jsp2.70105

**Published:** 2025-08-13

**Authors:** Alaina L. Falck, Erick O. Buko, Kayla L. Chase, Diana Pendleton, Katie McDermott, Olivia Kim, Suhail P. Parvaze, Alexandra R. Armstrong, Susan A. Arnold, Elizabeth W. Bradley, Arin M. Ellingson, Christopher P. Ober, Aaron Rendahl, Casey P. Johnson

**Affiliations:** ^1^ Department of Veterinary Clinical Sciences University of Minnesota Saint Paul Minnesota USA; ^2^ Center for Magnetic Resonance Research University of Minnesota Minneapolis Minnesota USA; ^3^ Veterinary Diagnostic Laboratory University of Minnesota Saint Paul Minnesota USA; ^4^ Department of Orthopedic Surgery University of Minnesota Minneapolis Minnesota USA; ^5^ Stem Cell Institute University of Minnesota Minneapolis Minnesota USA; ^6^ Divisions of Physical Therapy and Rehabilitation Science, Department of Family Medicine and Community Health University of Minnesota Minneapolis Minnesota USA; ^7^ Darkside Veterinary Imaging LLC Woodbury Minnesota USA; ^8^ Department of Veterinary Biomedical Sciences University of Minnesota Saint Paul Minnesota USA

**Keywords:** canine, degeneration, dog, intervertebral disc disease, magnetic resonance imaging, quantitative, spine

## Abstract

**Background:**

Client‐owned dogs presenting clinically with intervertebral disc disease (IVDD) are a potential comparative animal model to help advance the understanding of disc degeneration and its treatment. To utilize dog patients as a model, noninvasive imaging techniques are needed that can characterize subtle and progressive changes in disc health in longitudinal and treatment efficacy studies. The purpose of this study was to assess the sensitivity of quantitative MRI techniques in detecting disc degeneration in client‐owned, nonchondrodystrophic‐breed dogs.

**Methods:**

Thoracolumbar vertebral columns from the donated bodies of 15 dogs without a history of IVDD were imaged at 3T MRI. Quantitative MRI maps (T2, T2*, T1ρ, adiabatic T1ρ, adiabatic T2ρ, and ADC) were acquired axially for 10 discs (T11‐T12 to L7‐S1), and median values were measured in the nucleus pulposus and annulus fibrosus. Four disc health measures (Pfirrmann grade, histology score, water content, and glycosaminoglycan content) were evaluated for each disc. The quantitative MRI and disc health measures were compared using linear models, and partial correlations (*R*_partial_) were calculated.

**Results:**

Most dogs had both relatively healthy and degenerated discs as assessed by Pfirrmann grade and histology score. Quantitative MRI values in relatively healthy discs varied greatly between dogs but were similar across disc levels. In the nucleus pulposus, T2 relaxation times were moderately correlated with Pfirrmann grade (*R*_partial_ = −0.62; *p* < 0.0001), histology score (*R*_partial_ = −0.63; *p* < 0.0001), and water content (*R*_partial_ = +0.45; *p* < 0.0001), and weakly correlated with glycosaminoglycan content (*R*_partial_ = +0.31; *p* = 0.0047). T2, T2*, T1ρ, adiabatic T1ρ, and adiabatic T2ρ had similar relationships to the disc health measures in the nucleus pulposus. No notable relationships were observed with ADC or in the annulus fibrosus.

**Conclusions:**

Quantitative T2, T2*, T1ρ, adiabatic T1ρ, and adiabatic T2ρ relaxation time mapping techniques are similarly related to radiological and histological measures of disc health and water and glycosaminoglycan content in nonchondrodystrophic‐breed dogs.

## Graphical abstract

The purpose of this study was to assess the sensitivity of quantitative MRI techniques in detecting disc degeneration in client‐owned, nonchondrodystrophic‐breed dogs. Thoracolumbar vertebral columns from the donated bodies of 15 dogs without a history of IVDD were imaged ex vivo at 3T MRI, and quantitative MRI values of the discs were compared to radiological Pfirrmann grade, histology score, water content, and glycosaminoglycan content. We found that quantitative T2, T2*, T1ρ, adiabatic T1ρ, and adiabatic T2ρ relaxation time mapping techniques are similarly related to radiological and histological measures of disc health and water and glycosaminoglycan content.

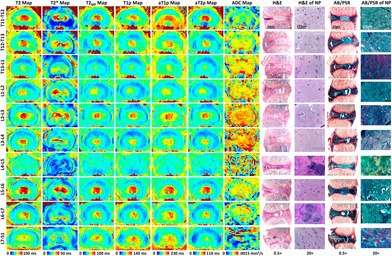

## 1 Introduction

Low back pain (LBP) is a leading cause of disability worldwide, affecting more than 600 million people and posing an enormous socioeconomic burden on society [1]. One of the main drivers of LBP, accounting for up to 45% of all cases, is intervertebral disc (IVD) degeneration [2]. In healthy spines, IVDs act as cushions between the vertebrae to help resist normal compressive mechanical forces, but when degenerative changes start to occur, the structural integrity and load‐bearing capacity of IVDs become compromised. Complex biological and biomechanical factors trigger a degenerative cascade in the IVD that includesdehydration of the disc driven by loss of glycosaminoglycans (GAG) in its inner gel‐like portion called the nucleus pulposus (NP); a reduction of type II collagen and an increase in type I collagen in the NP, resulting in its reduced elasticity; and the development of tears in the outer lamellar ring of the disc called the annulus fibrosus (AF) [3, 4]. Weakening of the IVDs can lead to LBP through a number of different mechanisms including neural in‐growth into the discs, disc herniation, spinal stenosis, and degenerative spondylolisthesis [4, 5].

Large animal models are critical to advancing our understanding of IVD degeneration and LBP and to investigating new treatments [6]. Most current large animal models of IVD degeneration use artificial means to induce disc injury (such as puncturing the NP with a needle), which does not replicate the natural pathogenesis of the disease. In contrast, naturally occurring IVD degeneration in dogs presents a unique opportunity to advance understanding and treatment of IVD degeneration and LBP [7]. IVD degeneration in dogs, commonly referred to as intervertebral disc disease (IVDD), is caused by similar inflammatory and degradative mechanisms to those that drive human IVD degeneration [8, 9], with client‐owned dogs being clinically similar to humans in terms of clinical signs, diagnostics, treatment, histology, and biochemistry of disc degeneration [10, 11]. Nonchondrodystrophic (NCD) breed dogs may be particularly useful to study the more gradual, chronic degenerative process seen in older adults and to evaluate notochordal‐rich discs to inform regenerative therapies [7, 11, 12, 13], as these breeds have persistent notochordal cells that preserve disc health for longer than chondrodystrophic (CD) breeds and are therefore more likely to experience chronic IVDD and disc protrusion at 6 years of age or older [14, 15]. Due to (i) the resemblance in the degenerative process between dogs and humans, (ii) their relatively comparably sized IVDs that reduce issues of scaling up from smaller models like rodents, and (iii) the ample veterinary patient population available for study and treatment trials, dogs are receiving increasing attention as models for human IVD degeneration and other spinal disorders, with potential dual species benefit [7, 11, 16, 17, 18, 19].

Clinical diagnosis of IVD degeneration in both humans and client‐owned dogs relies on T2‐weighted magnetic resonance imaging (MRI), which is the leading imaging modality to detect changes in degenerated discs such as height loss and reduced water signal intensity. T2‐weighted images are typically clinically evaluated using the radiological Pfirrmann grading system, which uses signal intensity and geometric features to identify problematic discs [20, 21, 22]. However, this qualitative grading method has limited ability to detect early and/or progressive changes in disc health [23] and to distinguish clinical from silent degenerative changes [22, 24]. This limits clinicians' ability to identify disease‐causing discs and to assess indications for and effectiveness of various treatment options [25]. Consequently, alternative imaging techniques are needed to detect and define early degenerative changes and to aid in the evaluation of new therapies.

Quantitative MRI techniques can provide more specific information about disc health than traditional, qualitative T2‐weighted MRI, and they are particularly valuable in longitudinal in vivo studies to track disc changes over time. In humans, quantification of MRI T2, T2*, and T1ρ relaxation times and apparent diffusion coefficient (ADC) has been shown to be sensitive surrogates of the biochemical composition of IVDs [26, 27, 28, 29, 30]. In studies of human discs, it has been found that T2, T1ρ, T2*, and ADC are all sensitive to changes in water content and the extracellular matrix composition of the NP, with T1ρ potentially providing additional sensitivity to early changes in GAG content [26, 27, 28]. Other quantitative MRI methods, such as adiabatic T1ρ (aT1ρ) and adiabatic T2ρ (aT2ρ) relaxation time mapping, may also provide advantages for imaging intervertebral discs; for example, aT1ρ and aT2ρ can potentially provide unique sensitivities that are a combination of T1, T2, and T1ρ relaxation processes [31, 32, 33]. As in humans, quantitative MRI techniques may also be useful to evaluate disc health and response to treatments in dogs, but their evaluation in dogs to date has been limited [16, 34, 35, 36, 37]. Comparison of these different quantitative MRI measures can help determine which methods are most sensitive and suitable as indirect disc health measures, such as water and GAG content, in dogs.

The purpose of the present study was to investigate the sensitivities of T2, T2*, T1ρ, aT1ρ, aT2ρ, and ADC mappings in detecting degenerative changes in thoracolumbar IVDs in a sample of client‐owned, NCD‐breed dogs. We sought to determine how these quantitative MRI measures in the NP and AF compare across different dogs (of varying breed, age, and sex), disc levels (T11‐T12 to L7‐S1), and measures of disc health (radiological Pfirrmann grade, histology score, water content, and GAG content). Thoracolumbar vertebral column segments from donated dog bodies without a clinical history of IVDD were imaged ex vivo at 3T MRI, and the discs were then sectioned for histological grading and biochemical assays. We hypothesized that: (i) the dogs would have a range of disc health, including degenerated discs; and (ii) the quantitative MRI techniques would provide noninvasive, correlative measures of disc health. This study is an important step toward using quantitative MRI measures to noninvasively evaluate disc health in client‐owned dogs with IVDD to advance both dog and human health through comparative studies.

## 2 Materials and Methods

### 2.1 Vertebral Column Specimens

This study was exempt from IACUC review as it received tissues from dog cadavers. The cadavers were collected through the University of Minnesota's veterinary body donation program at the Veterinary Medical Center. These were client‐owned dogs, whose owners elected for humane euthanasia in consultation with their attending veterinarian. Euthanasia was performed following AVMA guidelines and ethical standards, and the clients voluntarily released their dogs' bodies for scientific research. For this study, thoracolumbar vertebral column specimens spanning vertebrae T11 to S1 were collected from 15 of the donated bodies of dogs identified as NCD breeds. The dogs had varying signalments (Table 1), and none had a known clinical history of IVDD. It is of note that genetic testing was not performed to determine whether the dogs were purebred or were in fact NCD; thus, it is possible that some dogs had chondrodystrophy. The dogs included 9 males and 6 females, and most were elderly, with a mean age of 11.0 ± 2.9 years (range = 6.8–15.5 years). The donated bodies and harvested vertebral column specimens were refrigerated at 4°C until the time of MRI (0–4 days postmortem).

**TABLE 1:** Signalments of dogs included in the study.

Dog	Breed	Sex	Age (years)	Weight (kg)	Spay/neuter status	Days imaged postmortem
1	Rottweiler	F	6.8	39	Spayed	2
2	Vizsla	F	10.2	12	Spayed	1
3	Canaan	F	11.0	NA	Spayed	2
4	German Shepherd	F	11.0	33	Spayed	1
5	German Shepherd	F	13.4	32	Spayed	0
6	Labrador Retriever	F	15.1	NA	Spayed	2
7	Golden Retriever	M	5.0	46	Intact	1
8	Boxer	M	8.5	30	Neutered	1
9	Australian Cattle Dog	M	9.2	12	Intact	1
10	Labrador Retriever	M	10.4	12	Neutered	4
11	British Labrador Retriever	M	10.5	26	Neutered	2
12	Border Collie	M	12.3	30	Neutered	2
13	Labrador Retriever	M	13.0	NA	N/A	3
14	Airedale Terrier	M	13.3	35	Neutered	4
15	Australian Shepherd	M	15.5	29	Neutered	2

### 2.2 Magnetic Resonance Imaging

Each of the 15 vertebral column specimens was imaged ex vivo using a 3T MRI scanner (MAGNETOM Prisma; Siemens Healthcare) and vendor‐provided spine and/or flex matrix coils. The MRI protocol and imaging parameters are shown in Table 2. First, conventional sagittal and coronal T2‐weighted images were acquired for Pfirrmann grading. Second, the quantitative MRI maps (T2, T2*, T1ρ, aT1ρ, aT2ρ, and ADC) were acquired at a single axial slice for each of the 10 discs (T11‐T12 to L7‐S1). T2 maps were acquired using both a clinically available multi‐slice multi‐echo (MSME) spin echo sequence (T2) and a research magnetization‐prepared turbo spin echo sequence (T2_MP_); T2* maps were acquired using a clinically available MSME gradient echo sequence; T2_MP_, T1ρ, aT1ρ, and aT2ρ maps were acquired using the same research magnetization‐prepared turbo spin echo sequence with different preparation pulses; and ADC maps were acquired using a clinically available readout‐segmented diffusion‐weighted imaging (RESOLVE) sequence. Immediately following the MRI exam, the vertebral columns were frozen at −20°C and stored for up to 7 months until processing for histology and biochemical assays. In total, 150 discs (10 discs from 15 dogs) were imaged and analyzed using MRI.

**TABLE 2:** 3T MRI scan parameters.

	T2‐weighted	T2‐weighted	T2 map	T2* map	T2_MP_ map	T1ρ map	aT1ρ map	aT2ρ map	ADC
Sequence type	2D TSE	2D TSE	2D MSME spin echo	2D MSME GRE	2D MP TSE	2D MP TSE	2D MP TSE	2D MP TSE	RESOLVE DWI
Orientation	Sagittal	Coronal	Axial	Axial	Axial	Axial	Axial	Axial	Axial
Field of view (cm)	40 × 18	40 × 24	20 × 12	20 × 12	20 × 9	20 × 9	20 × 9	20 × 9	20 × 12
Resolution (mm)	0.78 × 0.78	0.78 × 0.78	0.52 × 0.52	0.52 × 0.52	0.52 × 0.52	0.52 × 0.52	0.52 × 0.52	0.52 × 0.52	1.1 × 1.1
Slices/thickness/gap (mm)	24/2.0/0	21/3.0/0.6	10/2.0/—	10/2.0/—	10/2.0/—	10/2.0/—	10/2.0/—	10/2.0/—	10/2.0/—
TR/TEs (ms)	3220/84	5000/104	2600/[10.7, 21.4, 32.1, …, 85.6]	1500/[3.9, 10.2, 16.6, …, 73.5]	2960/13	2960/13	2960/13	2960/13	2500/[54, 90]
Flip angle (°)	80/160	75/150	90/180	60	90/180	90/180	90/180	90/180	90/180
Bandwidth (Hz/px)	257	574	228	260	130	130	130	130	604
Fat saturation	—	—	—	—	—	—	—	—	Yes
Acceleration	—	—	—	—	—	—	—	—	*R* = 2 GRAPPA
Turbo or EPI factor	17	19	—	—	26	26	26	26	57
Preparation times (ms)	—	—	—	—	0, 20, 40, 60, 80	0, 20, 40, 60, 80	0, 24, 48, 72, 96	0, 24, 48, 72, 96	—
Preparation type	—	—	—	—	MLEV4	300 Hz spin lock pulse	6 ms HS1 pulse; 1000 Hz max	6 ms HS1 pulse; 1000 Hz max	3 directions; *b* = 0, 500, 1000 s/mm^2^
Scan time (min)	3	5.5	10	6	20	20	20	20	7.5

Abbreviations: DWI, diffusion‐weighted imaging; EPI, echo planar imaging; GRAPPA, generalized autocalibrating partially parallel acquisitions; GRE, gradient echo; HS1, hyperbolic secant pulse; MLEV4, Malcolm‐Levitt phase cycling pulse; MP, magnetization prepared; MSME, multi‐slice multi‐echo; RESOLVE, readout segmentation of long variable echo‐trains; TE, echo time; TR, repetition time; TSE, turbo spin echo.

The quantitative MRI maps were generated offline by fitting their respective weighted images to a mono‐exponential signal decay model in MATLAB (MathWorks; Natick, MA). Specifically, the equation used for fitting the T2 (and similarly the other) relaxation time maps was $S\left(\mathrm{TE}\right)=S\left(0\right)˙e^{-\mathrm{TE}/T2}$, and the equation used for fitting the ADC maps was $S\left(b\right)=S\left(0\right)˙e^{-b˙\mathrm{ADC}}$. For MSME T2 mapping, the first echo image was excluded from the fit to reduce the influence of stimulated echoes. The T2‐weighted images of the MSME T2 map acquisition were used to manually segment the disc into three regions of interest (ROIs) using ITK‐SNAP (www.itksnap.org) [38]: (i) NP; (ii) ventral AF (vAF); and (iii) dorsal AF (dAF). For segmentation, a central region was defined as the NP ROI, using the contrast difference between the NP and AF to approximate the NP boundary (in the absence of a clear boundary, an elliptical ROI was used). The AF was divided into four quadrants using orthogonal lines intersecting in the middle of the disc to define the vAF and dAF ROIs. Using MATLAB, all of the quantitative maps were spatially co‐registered to the T2‐weighted images based on DICOM header positioning information, and the median T2, T2*, T2_MP_, T1ρ, aT1ρ, aT2ρ, and ADC values within each of the three ROIs were calculated for each disc. Select ROI measurements for the ADC maps were excluded from the data analysis due to insufficient image quality. The Pfirrmann grade of each disc was determined by a board‐certified veterinary radiologist (C.P.O.) using the conventional sagittal and coronal T2‐weighted MRI images [20].

### 2.3 Histology

Each of the 15 frozen vertebral columns was bisected along the sagittal midline of the spine using a bandsaw. One half of each vertebral column was used for histology, and the other half was used to collect samples for the biochemical assays. For histology, and while the specimens were still frozen, each of the 10 discs (T11‐T12 to L7‐S1) was separated with endplates attached, cut into a 3–5 mm thick slab, and immediately fixed in 10% neutral buffered formalin. Each fixed specimen was subsequently decalcified in 10% ethylenediaminetetraacetic acid for approximately 4 months. The decalcified discs with attached endplates were then processed into paraffin blocks and cut into 5‐μm‐thick slides for staining using hematoxylin and eosin (H&E) and Alcian blue/Picrosirius red (AB/PSR) [6]. The degree of disc degeneration was assessed by a veterinary pathology resident (K.M.) who was blinded to the dog signalments using all nine scoring categories of the histological grading scheme defined by Bergknut et al. [39] Although a very similar, cross‐species grading scheme was recently proposed by Lee et al. [6] we opted to use the more established Bergknut grading scheme given its specific focus on IVDD in dogs and its concisely and clearly defined scoring categories relevant to this study. Note that while freezing may have slightly impacted histological quality, the grading is based on major morphological and compositional differences between the discs that would not result from a single freeze–thaw cycle. In total, 146 discs (9–10 discs from 15 dogs) were scored using histology (one T11/T12 disc was not sampled, and three discs were excluded due to loss of NP tissue during processing).

### 2.4 Biochemical Assays

The other half of the bisected, frozen vertebral column specimens were used to extract tissue samples for biochemical assays for a subset of 10 of the specimens (i.e., 99 discs, as one T11/T12 disc was not sampled; the biochemical tests for the initial five vertebral columns used an earlier version of the protocol that was found to be unreliable, and thus these samples were excluded from the analysis). The samples were collected while the discs were still frozen to minimize any dehydration of the tissue. Two samples were taken: one from the NP and one from the ventral portion of the AF (vAF). The dorsal AF (dAF) was often too small to obtain a sufficiently large tissue sample for analysis; thus, dAF samples were not included in the biochemical assay tests. The samples were cut out of the sagittal sections using a clean scalpel to minimize contamination from other tissues, and they were immediately sealed in individual vials and kept frozen for storage. For biochemical testing, the samples were thawed and assessed for water weight and sulfated GAG content. Specifically, the tissue samples were weighed in their vials (to ensure no loss of water content) before and after 20 h of dehydration in a 65°C oven. Water content was calculated as the difference in tissue sample weight before and after dehydration as a percentage of wet weight. The wet and dry weights of the samples were on average 55 ± 28 and 19 ± 12 mg, respectively. The samples were then digested with Pronase (Sigma‐Aldrich) and Collagenase Type 2 (Worthington Biochemical) enzymes and diluted either 1:100, 1:200, or 1:400 with PBS according to the original sample weight [40, 41]. The diluted samples were analyzed using a 1,9‐dimethylmethylene blue sGAG kit (Chondrex Inc.). Transmittance readings were obtained using a spectrophotometer at 620 nm. GAG content (i.e., GAG concentration normalized by dry weight) was measured by converting measured transmittance values to absorbance values, calculating GAG concentrations via regression analysis with known standard concentrations (ranging from 0 to 50 μg/mL), and multiplying by each sample's dilution factor.

### 2.5 Statistical Analysis

We first evaluated the disc health measures (Pfirrmann grade, histology score, water content, and GAG content) and their relationships to each other. We qualitatively assessed the distribution of the disc health measures across the different dogs and discs to determine (i) whether our sample of dogs had degenerated discs and (ii) whether any disc degeneration tended to occur at specific disc levels. We then quantitatively assessed how strongly the disc health measures were related to each other using Pearson's correlation coefficients (*r*).

Next, we evaluated whether the quantitative MRI values of relatively healthy discs (with Pfirrmann grades of 1 or 2) varied between different dogs or disc levels. This was to inform whether baseline (i.e., healthy) quantitative MRI values in the NP, vAF, and dAF regions of interest need to be considered on a per‐dog or per‐disc‐level basis. For this analysis, we first assessed differences between the dogs (considering all disc levels); and then differences between the disc levels (considering all dogs), using ANOVA with Tukey's post hoc test.

Lastly, we performed our primary evaluation to determine the relationships between the quantitative MRI values in the NP, vAF, and dAF (i.e., the predictor variables) and the disc health measures (i.e., the response variables). For this analysis, we used linear regression with “dog” and “disc level” as block effects to account for differences between the dogs and disc levels (this is equivalent to defining “dog” and “disc level” as random effects in a mixed‐effects model). We then measured the partial correlation (*R*_partial_) and slope (*m*) of the predictor variable terms (i.e., the quantitative MRI values), which inform the relationships between the predictor and response variables independent of the variance due to differences between the dogs and disc levels (conversely, the total correlation would include the variance due to differences between the dogs and disc levels). To provide a more practical measure of the predicted change in disc health with a change in quantitative MRI value, we also reported the change in quantitative MRI value (1/*m*) that corresponds to a unit change in the disc health measures.

All statistical analyses were performed using R statistical computing software (R version 4.3.1) [42]. The partial correlations were calculated using the rsq package [43], and results were displayed using the ggplot2 and corrplot packages [44, 45]. A *p* value of 0.05 was used to designate significance, except for the linear models, for which *p* < 0.0125 and *p* < 0.025 were considered statistically significant after Bonferroni correction for four and two disc health measures, respectively. Correlations were categorized as very weak (0–0.2), weak (0.2–0.4), moderate (0.4–0.6), strong (0.6–0.8), and very strong (0.8–1.0).

## 3 Results

### 3.1 Disc Health Measures

**FIGURE 1:**
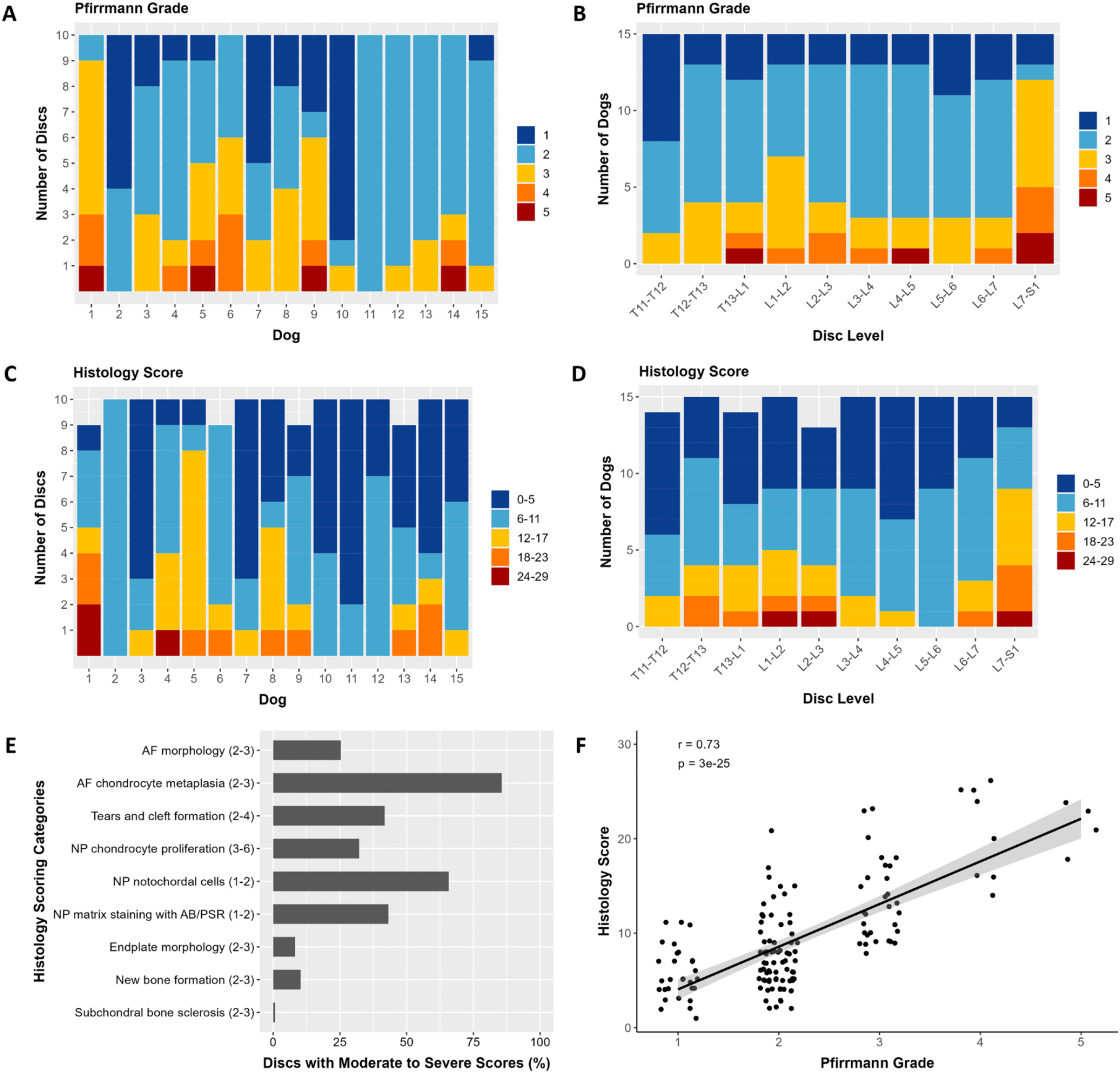
Radiological Pfirrmann grades and histology scores for each of the 9–10 discs of the 15 dogs. (A, C) Distribution of Pfirrmann grades and histology scores for each dog (combining data for all disc levels). Most dogs had a range of healthy and degenerated discs. (B, D) Distribution of Pfirrmann grades and histology scores for each disc level (combining data for all dogs). Degenerated discs were seen at all disc levels, most prevalently at the lumbosacral joint (L7‐S1). (E) Percentage of discs from all of the dogs that had moderate to severe scores for each of the histology categories. The score range that was considered moderate to severe for each category is indicated. (F) Correlation of Pfirrmann grade and histology score for all 146 discs.

The radiological and histological assessments revealed a range of intervertebral disc health within individual dogs across the 10 discs (T11‐T12 to L7‐S1) (Figure 1). A total of 150 discs (10 discs for 15 dogs) were evaluated with MRI, and 146 discs (9–10 discs for 15 dogs) were evaluated with histology. 13/15 of the dogs had both relatively healthy (Grades 1 and 2) and degenerated (Grades 3–5) discs as determined by the radiological Pfirrmann grade (Figure 1A). Out of 150 total discs, the number of discs with each Pfirrmann grade was 29 (Grade 1), 76 (Grade 2), 32 (Grade 3), 9 (Grade 4), and 4 (Grade 5). The distribution of Pfirrmann grades was similar at disc levels T11‐T12 to L6‐L7, but the discs of the lumbosacral joint (L7‐S1) were the most consistently degenerated, with only 3/15 dogs having relatively healthy discs at that level (Figure 1B). Across all disc levels, 15 discs had radiological evidence of at least moderate disc extrusion and 2 discs had evidence of at least moderate disc protrusion; 9 of these discs were at the L7‐S1 level. The dogs also had a range of disc histology scores (Figure 1C). The histology scores of individual discs ranged from 1 to 26 (out of a possible range of 0–29). Scores were generally similar across disc levels; L7‐S1 had the greatest proportion of degenerated discs (Figure 1D). Moderate to severe histology scores were most frequently noted in the following scoring categories as defined by Bergknut et al. [39]: AF morphology; AF chondrocyte metaplasia; tears and cleft formation; NP chondrocyte proliferation; NP notochordal cells; and NP matrix staining with AB/PSR (Figure 1E). The Pfirrmann grades and histology scores were strongly correlated across all 146 discs (*r* = +0.73; *p* < 0.0001) (Figure 1F).

**FIGURE 2:**
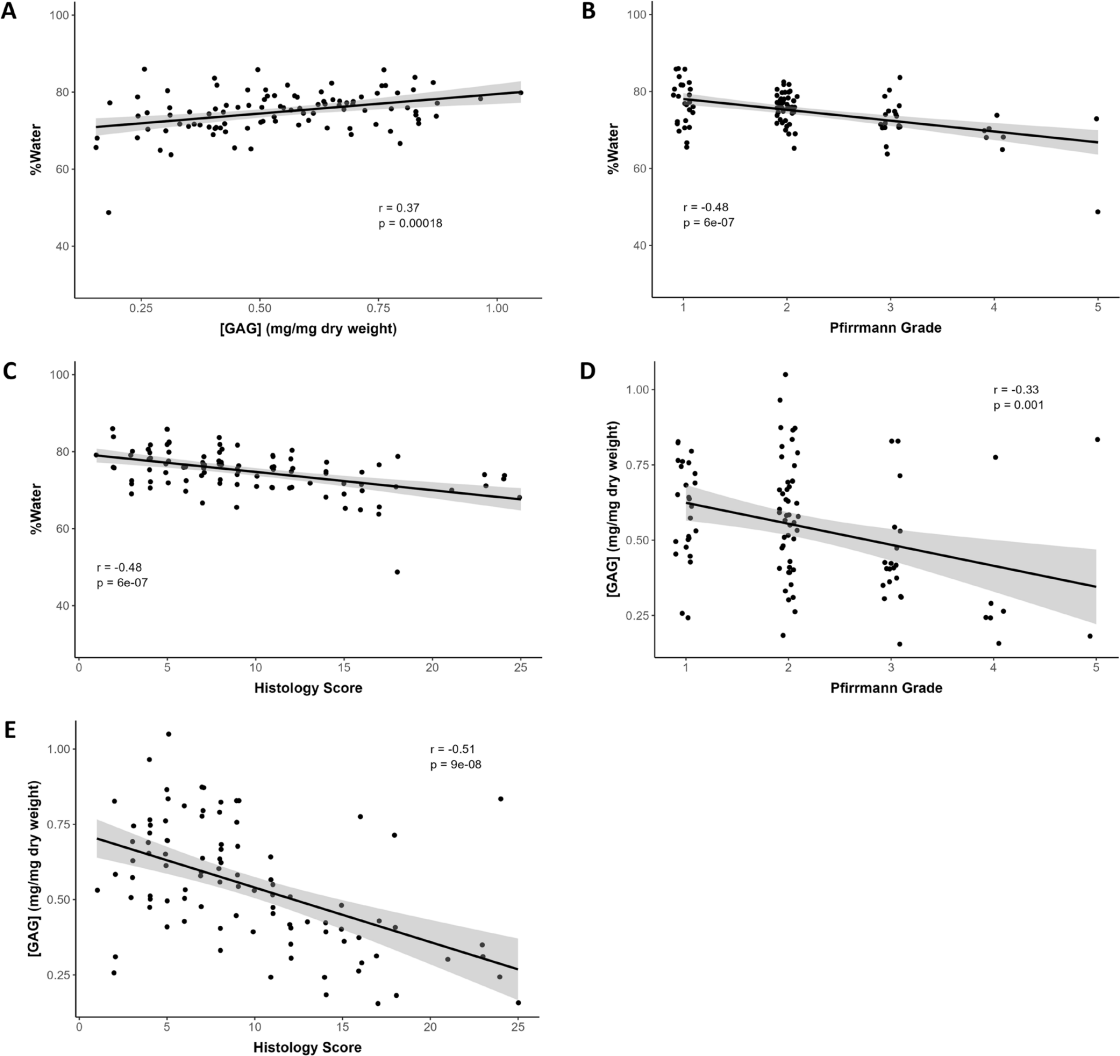
Correlation plots between water content, GAG content, Pfirrmann grade, and histology score in the nucleus pulposus (NP) for all discs from the subset of 10 dogs (99 total discs) assessed with biochemical assays. Water content in the NP was weakly and positively correlated with (A) GAG content, and it was moderately and negatively correlated with (B) Pfirrmann grade and (C) histology score. GAG content in the NP was weakly and negatively correlated with (D) Pfirrmann grade and moderately and negatively correlated with (E) histology score.

**FIGURE 3:**
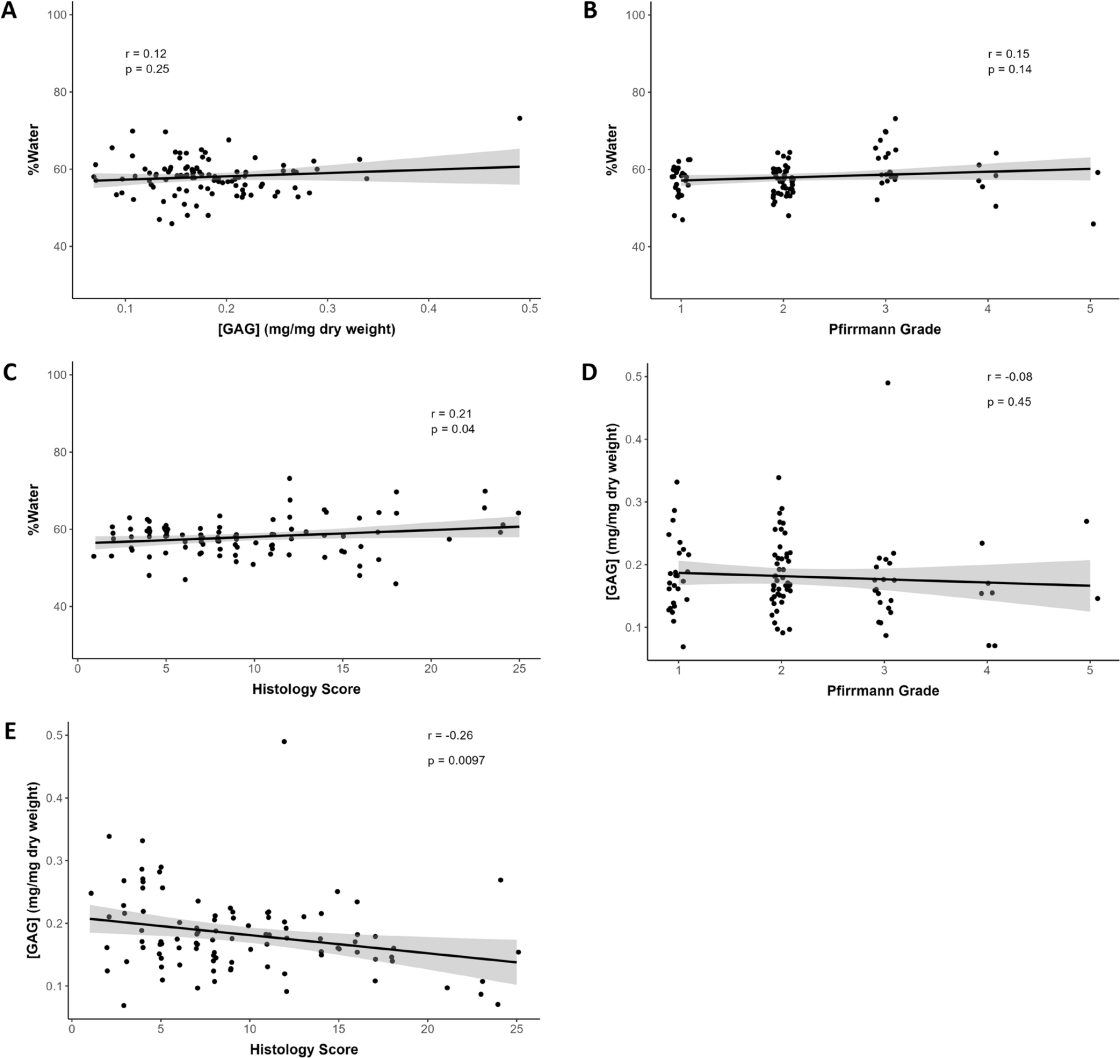
Correlation plots between water content, GAG content, Pfirrmann grade, and histology score in the ventral aspect of the annulus fibrosus (vAF) for all discs from the subset of 10 dogs (99 total discs) assessed with biochemical assays. Water content in the vAF was not correlated with (A) GAG content, (B) Pfirrmann grade, or (C) histology score. GAG content in the vAF was not correlated with (D) Pfirrmann grade, but it was weakly and negatively correlated with (E) histology score.

The biochemical assay assessments for the subset of 10 dogs evaluated (i.e., 99 discs total) identified a range of disc water and GAG content in the NP (Figure 2) and vAF (Figure 3). The water content of individual discs ranged from 48.7% to 86.0% with a mean of 74.9% ± 5.5% in the NP and from 45.9% to 73.2% with a mean of 58.0% ± 4.7% in the vAF. The GAG content of individual discs ranged from 0.15 to 1.05 mg/mg dry weight with a mean of 0.55 ± 0.20 mg/mg in the NP and from 0.07 to 0.49 mg/mg dry weight with a mean of 0.18 ± 0.06 mg/mg in the vAF. Water content in the NP was weakly and positively correlated with GAG content in the NP (*r* = +0.37; *p* = 0.0002; Figure 2A), moderately and negatively correlated with Pfirrmann grade (*r* = −0.48; *p* < 0.0001; Figure 2B), and moderately and negatively correlated with histology score (*r* = −0.48; *p* < 0.0001; Figure 2C). Water content in the vAF was not significantly correlated with GAG content (Figure 3A) or Pfirrmann grade (Figure 3B), but it was weakly and positively correlated with histology score (*r* = +0.21; *p* = 0.040; Figure 3C). GAG content in the NP was weakly and negatively correlated with Pfirrmann grade (*r* = −0.33; *p* = 0.0010; Figure 2D) and moderately and negatively correlated with histology score (*r* = −0.51; *p* < 0.0001; Figure 2E). GAG content in the vAF was not significantly correlated with Pfirrmann grade (Figure 3D), but it was weakly and negatively correlated with histology score (*r* = −0.26; *p* = 0.0097; Figure 3E).

### 3.2 Quantitative MRI Variations Between Dogs and Disc Levels

**FIGURE 4:**
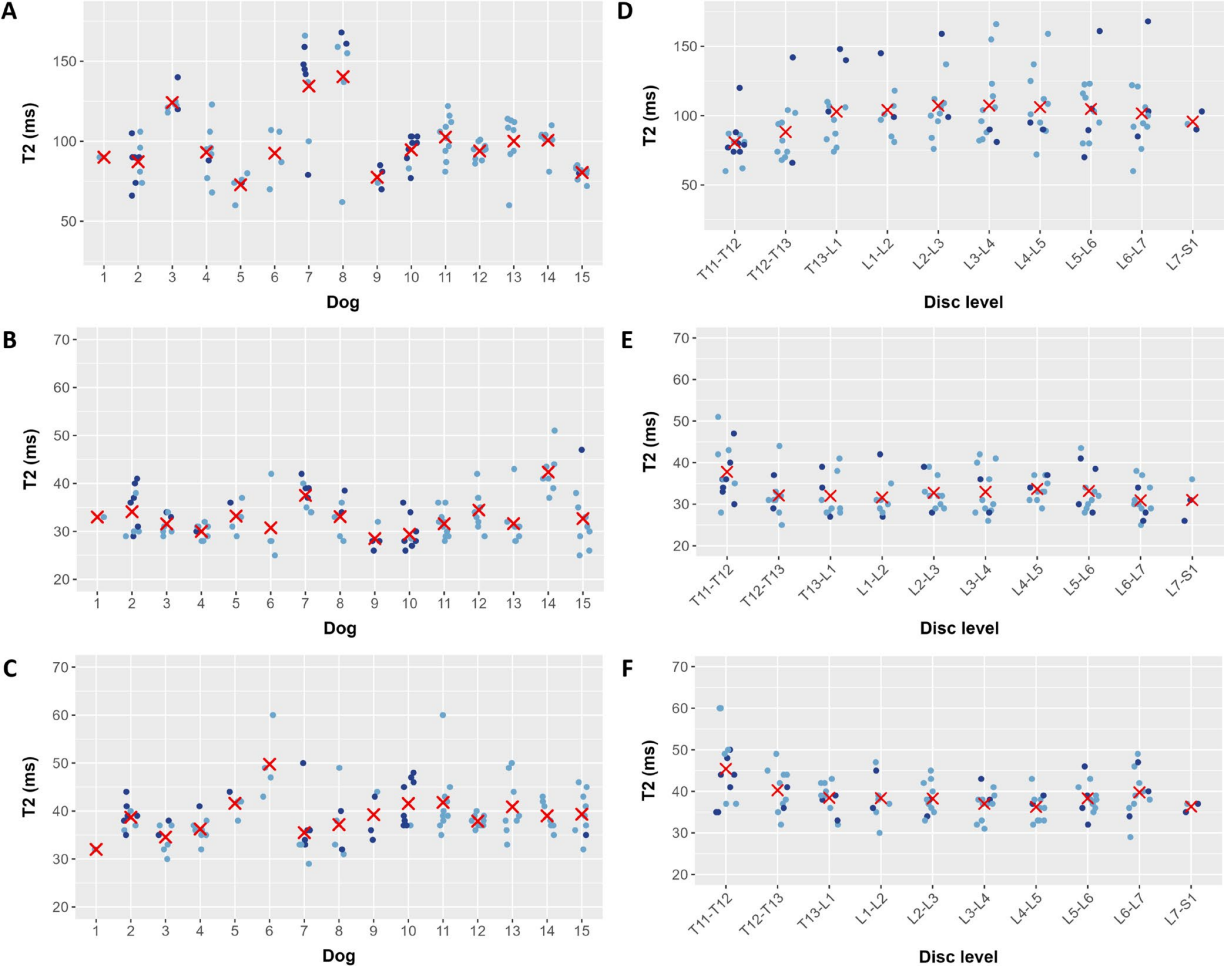
T2 relaxation times for relatively healthy discs (Pfirrmann grades 1 and 2). Values are plotted for each dog (including data for all disc levels) and each disc level (including data for all dogs) in the (A, D) nucleus pulposus (NP), (B, E) ventral aspect of the annulus fibrosus (vAF), and (C, F) dorsal aspect of the annulus fibrosus (dAF). Dark blue points indicate Pfirrmann grade 1, light blue points indicate Pfirrmann grade 2, and X's indicate mean values. Mean T2 relaxation times in the NP, vAF, and dAF varied greatly between the dogs, but they were relatively similar across disc levels.

To determine whether the quantitative MRI values in the NP, vAF, and dAF varied between dogs and disc levels, we restricted our comparison to relatively healthy discs with Pfirrmann grades of 1 or 2 (105 discs total, with at least one disc from each of the 15 dogs). The T2 relaxation times for each dog (across all disc levels) and for each disc level (across all dogs) are plotted in Figure 4. The T2 relaxation times varied greatly between dogs, with the mean T2 relaxation times for each dog ranging from 72.8 to 140.3 ms in the NP, 28.5 to 42.4 ms in the vAF, and 32.0 to 49.8 ms in the dAF. Using ANOVA with Tukey's post hoc test, 29 (27.6%), 15 (14.3%), and 8 (7.6%) of the 105 unique pairs of dogs had mean T2 relaxation times that were significantly different from each other in the NP, vAF, and dAF, respectively. There was relatively little variation in T2 relaxation times across disc levels in the NP, vAF, or dAF, and the differences were only significant using ANOVA in 1 (2.2%) and 5 (11.1%) of the 45 pairs of disc levels in the vAF and dAF, respectively. The other six quantitative MRI measures also varied to a similar degree between the dogs and disc levels; the number of pairs that were different for each quantitative MRI measure is tabulated in Table 3.

**TABLE 3:** Number of pairs of dogs and disc levels with significant differences in mean quantitative MRI values in each region of interest.

	T2	T2*	T2_MP_	T1ρ	aT1ρ	aT2ρ	ADC
*Pairs of dogs (out of 105)*							
NP	29	16	28	28	24	27	35
vAF	15	1	24	17	22	15	13
dAF	8	2	20	6	ns	ns	6
*Pairs of disc levels (out of 45)*							
NP	ns	ns	ns	ns	ns	ns	ns
vAF	1	ns	ns	2	1	1	ns
dAF	5	9	ns	2	6	ns	ns

Abbreviations: dAF, dorsal aspect of the annulus fibrosus; NP, nucleus pulposus; ns, ANOVA not significant (*p* > 0.05); vAF, ventral aspect of the annulus fibrosus.

### 3.3 Relationships Between Quantitative MRI and Disc Health Measures

**FIGURE 5:**
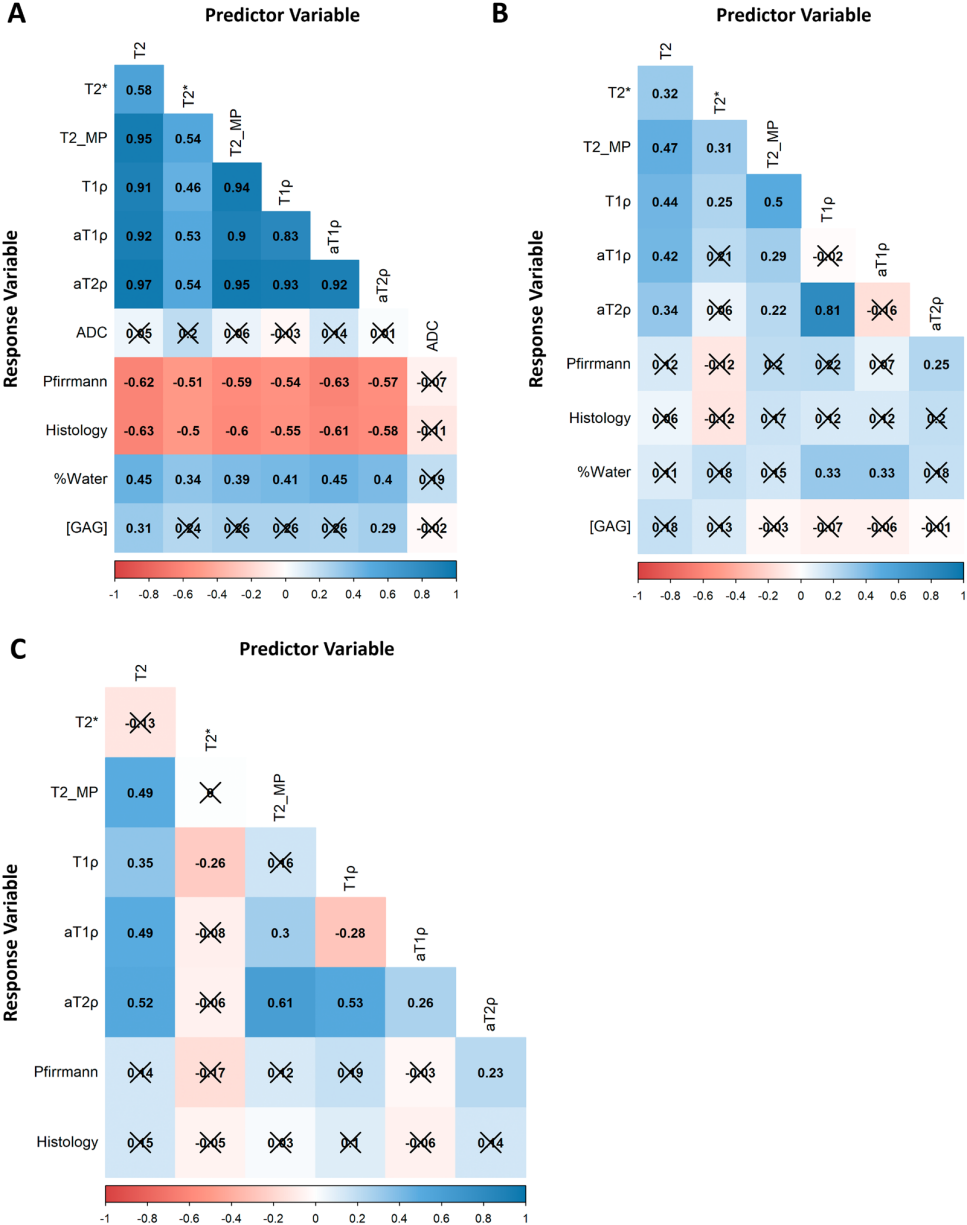
Partial correlations between the quantitative MRI and disc health measures in the (A) nucleus pulposus (NP), (B) ventral aspect of the annulus fibrosus (vAF), and (C) dorsal aspect of the annulus fibrosus (dAF) using linear models with “dog” and “disc level” as block effects. Crossed out values (×) are nonsignificant (*p* ≥ 0.0125 or *p* ≥ 0.025 with Bonferroni correction for four (A and B) or two (C) disc health measures, respectively). In the NP, the relaxation times were moderately and negatively correlated with Pfirrmann grade and histology score, and moderately and positively correlated with water content. T2 and aT2ρ were also weakly and positively correlated with GAG content. In the vAF and dAF, aT2ρ was weakly and positively correlated with Pfirrmann grade. Additionally, in the vAF, T1ρ and aT1ρ were weakly and positively correlated with water content. Additional details of these relationships are shown in Tables 4, 5, 6.

Using linear models with dog and disc level as block effects, we found significant relationships between the quantitative MRI and disc health measures in the NP but few in the vAF or dAF. Figure 5 plots the partial correlations (*R*_partial_) for each of the predictor quantitative MRI variables and each of the response variables (including the other quantitative MRI measures and the disc health measures) in the NP, vAF, and dAF. In the NP, all six relaxation times (T2, T2*, T2_MP_, T1ρ, aT1ρ, and aT2ρ) were significantly (*p* < 0.0125) and moderately (*R*_partial_ = 0.4–0.6) related to Pfirrmann grade (negative correlation), histology score (negative correlation), and water content (positive correlation). T2 and aT2ρ were significantly (*p* < 0.0125) and weakly (*R*_partial_ = 0.2–0.4) related to GAG content (positive correlation; the other four relaxation times had similar relationships to GAG content, but were nonsignificant after Bonferroni correction). Furthermore, we found that the six relaxation times were all correlated with each other, and there were no clear differences in the sensitivities of the relaxation times to the various measures of disc health. ADC did not have any significant relationships to the response variables. In the vAF and dAF, aT2ρ was significantly (*p* < 0.0125) and weakly (*R*_partial_ = 0.2 to 0.4) related to Pfirrmann grade (positive correlation). In the vAF, T1ρ and aT1ρ were significantly (*p* < 0.0125) and weakly (*R*_partial_ = 0.2 to 0.4) related to water content (positive correlation). No other significant relationships were identified between the quantitative MRI and disc health measures in the vAF and dAF.

Table 4 details, for the NP, the partial correlations (*R*_partial_), slopes (*m*), and *p* values for the relationships between the quantitative MRI measures and the disc health measures. For example, in the NP, we found that T2 relaxation timesdecreased 45.5 ms for a 1.0 grade increase in Pfirrmann grade; decreased 7.5 ms for a 1.0 point increase in histology score; decreased 12.9 ms for a 1% decrease in water content; and decreased 41.6 ms for a 0.1 mg/mg dry weight decrease in GAG content. Similar tables for the vAF and dAF are shown in Tables 5 and 6, respectively.

**TABLE 4:** Relationships between the quantitative MRI and disc health measures in the nucleus pulposus (NP) using linear regression models with dog and disc level as block effects.

	T2	T2*	T2_MP_	T1ρ	aT1ρ	aT2ρ	ADC
*Pfirrmann grade*							
*R*_partial_	−0.62	−0.51	−0.59	−0.54	−0.63	−0.57	−0.07
1/*m* (units/grade)	−45.5	−19.8	−52.5	−82.4	−94.2	−53.6	−2.13
*m* (grades/unit)	−0.022	−0.050	−0.019	−0.012	−0.011	−0.019	−0.47
95% CI	[−0.027, −0.017]	[−0.066, −0.035]	[−0.024, −0.014]	[−0.015, −0.009]	[−0.013, −0.008]	[−0.023, −0.014]	[−1.73, 0.79]
*p* value	< 0.0001*	< 0.0001*	< 0.0001*	< 0.0001*	< 0.0001*	< 0.0001*	0.46
*Histology score*							
*R*_partial_	−0.63	−0.50	−0.60	−0.55	−0.61	−0.58	−0.11
1/*m* (units/point)	−7.5	−3.3	−8.7	−13.5	−16.3	−8.8	−0.23
*m* (points/unit)	−0.134	−0.303	−0.116	−0.074	−0.061	−0.114	−4.43
95% CI	[−0.164, −0.104]	[−0.396, −0.209]	[−0.144, −0.087]	[−0.094, −0.054]	[−0.076, −0.047]	[−0.143, −0.085]	[−11.67, 2.81]
*p* value	< 0.0001*	< 0.0001*	< 0.0001*	< 0.0001*	< 0.0001*	< 0.0001*	0.23
*Water content*							
*R*_partial_	0.45	0.34	0.39	0.41	0.45	0.40	0.19
1/*m* (units/%)	12.9	6.1	15.7	21.1	25.5	15.6	0.13
*m* (%/unit)	0.077	0.164	0.064	0.047	0.039	0.064	7.81
95% CI	[0.043, 0.112]	[0.063, 0.266]	[0.030, 0.098]	[0.024, 0.071]	[0.022, 0.057]	[0.032, 0.097]	[−1.06, 16.69]
*p* value	< 0.0001*	0.0018*	0.0003*	0.0001*	< 0.0001*	0.0002*	0.83
*GAG content*							
*R*_partial_	0.31	0.24	0.26	0.26	0.26	0.29	−0.02
1/*m* (units/0.1 mg/mg dry weight)	41.6	19.3	52.0	73.3	98.7	49.0	−2.3
*m* (0.1 mg/mg dry weight/unit)	0.024	0.051	0.019	0.014	0.010	0.020	−0.431
95% CI	[0.008, 0.040]	[0.005, 0.099]	[0.003, 0.035]	[0.002, 0.025]	[0.002, 0.019]	[0.005, 0.036]	[−4.50, 3.63]
*p* value	0.0047*	0.032	0.019	0.018	0.021	0.0097*	0.83

Abbreviations: *m* = slopes of the relationship between the quantitative MRI and disc health measures (the units are ms for the relaxation times and 10^−3^ mm^2^/s for ADC); *R*_partial_ = partial correlations of the quantitative MRI and disc health measures.

* Statistically significant after Bonferroni correction for four disc health measures.

**TABLE 5:** Relationships between the quantitative MRI and disc health measures in the ventral aspect of the annulus fibrosus (vAF) using linear regression models with dog and disc level as block effects.

	T2	T2*	T2_MP_	T1ρ	aT1ρ	aT2ρ
*Pfirrmann grade*						
*R*_partial_	0.12	−0.12	0.20	0.22	0.07	0.25
1/*m* (ms/grade)	49.6	−32.8	30.6	92.6	221.8	58.0
*m* (grades/ms)	0.020	−0.031	0.033	0.011	0.005	0.017
95% CI	[−0.008, 0.049]	[−0.075, 0.014]	[0.005, 0.061]	[0.002, 0.019]	[−0.006, 0.015]	[0.005, 0.029]
*p* value	0.16	0.18	0.023	0.015	0.41	0.0047*
*Histology score*						
*R*_partial_	0.06	−0.12	0.17	0.12	0.12	0.20
1/*m* (ms/point)	18.2	−5.4	6.2	27.1	22.2	12.4
*m* (points/ms)	0.055	−0.184	0.160	0.037	0.045	0.080
95% CI	[−0.117, 0.226]	[−0.453, 0.086]	[−0.010, 0.330]	[−0.016, 0.090]	[−0.020, 0.110]	[0.009, 0.153]
*p* value	0.53	0.18	0.065	0.17	0.17	0.028
*Water content*						
*R*_partial_	0.11	0.18	0.15	0.33	0.33	0.18
1/*m* (ms/%)	12.8	4.4	7.1	4.7	9.7	6.1
*m* (%/ms)	0.078	0.225	0.141	0.212	0.103	0.164
95% CI	[−0.075, 0.231]	[−0.044, 0.495]	[−0.074, 0.356]	[0.076, 0.347]	[0.036, 0.169]	[−0.037, 0.366]
*p* value	0.31	0.10	0.19	0.0027*	0.0028*	0.11
*GAG content*						
*R*_partial_	0.18	0.13	−0.03	−0.07	−0.06	−0.01
1/*m* (ms/0.1 mg/mg dry weight)	49.0	39.1	−194.0	−138.0	−312.8	−561.5
*m* (0.1 mg/mg dry weight/ms)	0.020	0.026	−0.005	−0.007	−0.003	−0.002
95% CI	[−0.004, 0.045]	[−0.018, 0.069]	[−0.040, 0.030]	[−0.030, 0.016]	[−0.014, 0.008]	[−0.035, 0.031]
*p* value	0.10	0.25	0.77	0.53	0.57	0.91

Abbreviations: *m*, slopes of the relationship between the quantitative MRI and disc health measures; *R*_partial_, partial correlations of the quantitative MRI and disc health measures.

* Statistically significant after Bonferroni correction for four disc health measures.

**TABLE 6:** Relationships between the quantitative MRI and disc health measures in the dorsal aspect of the annulus fibrosus (dAF) using linear regression models with dog and disc level as block effects.

	T2	T2*	T2_MP_	T1ρ	aT1ρ	aT2ρ
*Pfirrmann grade*						
*R*_partial_	0.14	−0.17	0.12	0.19	−0.03	0.23
1/*m* (ms/grade)	48.6	−26.6	68.3	247.8	−660.0	30.1
*m* (grades/unit)	0.021	−0.038	0.015	0.004	−0.002	0.033
95% CI	[−0.005, 0.046]	[−0.077, 0.002]	[−0.006, 0.035]	[0.000, 0.008]	[−0.010, 0.007]	[0.008, 0.058]
*p* value	0.11	0.062	0.16	0.034	0.71	0.0094*
*Histology score*						
*R*_partial_	0.15	−0.05	0.03	0.10	−0.06	0.14
1/*m* (ms/point)	7.6	−14.4	48.1	75.9	−57.9	8.0
*m* (points/unit)	0.131	−0.069	0.021	0.013	−0.017	0.125
95% CI	[−0.026, 0.288]	[−0.313, 0.175]	[−0.105, 0.147]	[−0.010, 0.036]	[−0.066, 0.032]	[−0.029, 0.278]
*p* value	0.10	0.58	0.74	0.25	0.49	0.11

Abbreviations: *m*, slopes of the relationship between the quantitative MRI and disc health measures; *R*_partial_ = partial correlations of the quantitative MRI and disc health measures.

* Statistically significant after Bonferroni correction for two disc health measures.

**FIGURE 6:**
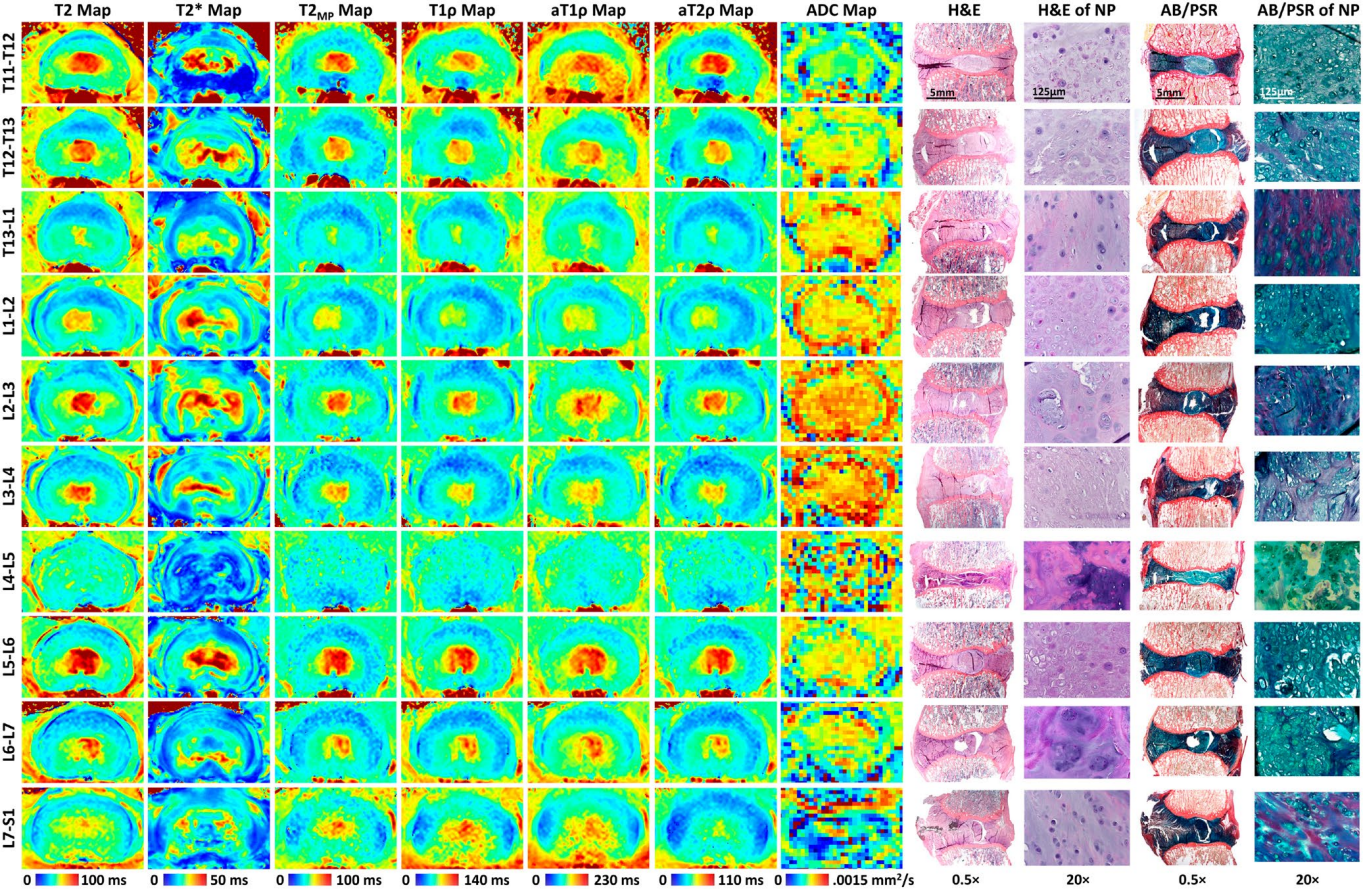
Quantitative MRI maps and H&E‐ and AB/PSR‐stained histological sections for each of the 10 discs for a representative dog (Dog #5). This dog had a range of disc health across levels, with both relatively healthy discs (Pfirrmann grade 1: T11‐T12; Pfirrmann grade 2: T12‐T13, L2‐L3, L5‐L6, and L6‐L7) and degenerated discs (Pfirrmann grade 3: L1‐L2, L3‐L4, L7‐S1; Pfirrmann grade 4: T13‐L1; Pfirrmann grade 5: L4‐L5). Histologically, these degenerated discs tended to have fewer notochordal cells in the NP, a greater number of proliferative chondrocytes in the NP, and/or greater degradation of the AF including the presence of tears and clefts. The relaxation time values were generally decreased in the nucleus pulposus (NP) of the degenerated discs, whereas ADC values were less reliable. Relaxation times in the annulus fibrosus (AF) were similar in both healthy and degenerated discs. While the T2, T2_MP_, T1ρ, adiabatic T1ρ (aT1ρ), and adiabatic T2ρ (aT2ρ) relaxation time maps had very similar appearances and changes across disc levels, T2* had a more unique appearance with a greater degree of signal heterogeneity in the NP.

To illustrate these relationships in a representative dog, all of the acquired quantitative MRI maps and H&E‐ and AB/PSR‐stained photomicrographs from one of the vertebral columns (Dog #5) are shown in Figure 6. Similar images from two other dogs (Dogs #6 and #13) are included as Figures S1 and S2. The T2, T2_MP_, T1ρ, aT1ρ, and aT2ρ relaxation time maps all had similar variations across the disc levels and identified both healthy discs (with high relaxation times in the NP) and degenerated discs (with low relaxation times in the NP). The T2* relaxation time maps had a distinct appearance, but they also showed a reduction in relaxation times in the degenerated NPs. ADC maps did not follow a clear trend across the discs and were of low quality. Furthermore, relaxation times showed little variation in the AF across the discs. In the photomicrographs, the healthy discs typically had a greater prevalence of notochordal cells in the NP and limited injury to the AF. Conversely, the degenerated discs often had fewer notochordal cells and a greater number of proliferative chondrocytes in the NP, along with evidence of tears and damage in the AF. The quantitative MRI maps generally reflect these compositional changes, with lower relaxation times in the NPs of the degenerated discs. However, some discs appeared healthy on MRI but had higher scores than expected histologically. These differences between the healthy and degenerated discs are representative of the general findings across all examined dogs.

## 4 Discussion

Our study provides a detailed characterization of the relationships between quantitative MRI measures and radiological, histological, and biochemical disc health measures in a client‐owned, general dog population. We found that T2, T2*, T1ρ, aT1ρ, and aT2ρ relaxation times were: (i) varied between dogs; (ii) similar across disc levels; and (iii) significantly related to disc health measures in the NP, including Pfirrmann grade, histology score, water content, and (to a lesser degree) GAG content. There were no notable differences in the relationships between the relaxation times. Although T2* and ADC had more unique responses, they were overall less strongly correlated with the disc health measures. These findings support that relaxation time mapping can be used as a noninvasive means to characterize disc health when controlling for differences between dogs.

Our study supports that NCD‐breed dogs have a high prevalence of subclinical IVDD. In our sampling of 15 companion, NCD‐breed dogs without a known history of IVDD, we found that most dogs had a range of disc health in the thoracolumbar spine, including both relatively healthy (Pfirrmann grades 1 and 2) and degenerated (Pfirrmann grades 3 to 5) discs. This is in agreement with a prior study by Bouhsina et al. that identified a similar distribution of Pfirrmann grades in dogs without a history of IVDD, with ~2/3 of discs having Pfirrmann grades 1 or 2 and ~1/3 of discs having Pfirrmann grades 3–4 [37]. However, our population had more Pfirrmann grade 2 versus 1 discs (whereas Bouhsina et al. reported the opposite), which may relate to the fact that our population of dogs was older (mean age 11.0 vs. 7.2 years) [21]. The fact that the individual dogs in our study often had both relatively healthy and degenerated discs supports that the moderate to severe disc degeneration is not only due to a gradual aging process (which may be expected to affect all disc levels similarly, as has been observed in sheep [46]). Rather, it is reflective of an accelerated disease process (which may be caused and perpetuated by numerous factors, including biomechanical instability and intervertebral disc dysfunction [7]) occurring at nonspecific disc levels that are not limited to sites predisposed to disc degeneration (such as the lumbosacral joint [47]). In addition to the comparative relevance of IVDD in NCD‐breed dogs to degenerative disc disease in humans [7], having dogs present with a range of disc health may be useful as a means to normalize quantitative MRI values across different dogs (i.e., there is an opportunity for intra‐dog disc comparisons). Also of note is that our identified relationships between Pfirrmann grade, histological score, water content, and GAG content agree with the prior literature in dogs, which supports the validity of our disc health assessments [7, 8, 39, 48].

T2 relaxation times were related to disc health measures in the dogs in a similar way to that shown in humans. In the NP, T2 relaxation times were negatively correlated with Pfirrmann grade (*R*_partial_ = −0.62) and histology score (*R*_partial_ = −0.55) and positively correlated with water content (*R*_partial_ = +0.45) and GAG content (*R*_partial_ = +0.31), consistent with dehydration and structural changes that occur in IVDs with degeneration. These trends also mimicked those found in two experimentally induced dog IVDD studies [34, 35] and in human studies [28]. Particularly promising is the correlation of T2 with histology, as histological changes are often considered the gold standard for disc degeneration research in both dogs and humans [39, 49, 50] and can provide a more precise measure of disc health than Pfirrmann grade. Since histology score cannot be measured in vivo, an ability to use T2 as an approximation of histology score would allow us to detect and interpret earlier and/or more subtle changes in disc health. For example, we determined that a 7.5 ms decrease in T2 corresponds on average to a 1.0 point increase in histology score. More studies are needed to translate this type of modeling to a clinical setting, but the associations we found support the feasibility of this endeavor. Notably, we found T2 relaxation times to be more strongly related to water content than GAG content in the NP, which supports that water content is the dominant contributor to the T2 relaxation time changes.

There were no clear differences in the relationships of the six relaxation time mapping methods to the various measures of disc health. T2_MP_, T1ρ, aT1ρ, and aT2ρ were all acquired using an identical pulse sequence but with different preparation pulses, allowing for a direct comparison of whether spin‐lock preparations provide any unique sensitivity to the measures of disc health. We found that these four relaxation times were very strongly correlated with each other in the NP (*R*_partial_ = +0.83 to +0.95) and had very similar correlations to Pfirrmann grade (*R*_partial_ = 0.54 to −0.63), histology score (*R*_partial_ = −0.55 to −0.63), water content (*R*_partial_ = +0.39 to +0.45), and GAG content (*R*_partial_ = +0.26 to +0.31), indicating that they did not provide any meaningfully different information about disc health. We had anticipated that T1ρ and aT1ρ may be more strongly correlated to GAG content than T2, but we did not find this to be the case. Note that this does not imply that these specific spin‐lock preparations are insensitive to GAG content, but rather that either the effect was too small, the concentration of GAG was too low, and/or the GAG measurements were too variable to be able to generate a measurable difference in sensitivity compared to T2. Although T2* and ADC showed more distinct responses than the other quantitative MRI measures, these differences were at least in part due to the technical challenges presented by these techniques. T2* is sensitive to magnetic susceptibility differences, which may be caused by mineralization in the disc or susceptibility artifacts from the nearby bony endplates. Thus, T2* may be more difficult to reliably quantify than T2 and other spin‐echo‐based measures that correct for the effects of local magnetic field inhomogeneities. On the other hand, T2* mapping may prove to be a useful, complementary technique to T2 mapping on account of its unique sensitivities, but this requires further investigation and validation. ADC mapping suffers from relatively poor spatial resolution as well as image distortions, which contributed to relatively poor quality and variability in the ADC maps. Overall, given the lack of apparent advantage to using any of the other quantitative MRI techniques over T2 relaxation time mapping, it may be most valuable to concentrate on T2 and T2* mapping in future quantitative MRI investigations of dog disc health at a 3T field strength.

The differences we observed between dogs in the quantitative MRI values of relatively healthy discs (Pfirrmann grades 1 and 2) may be driven by several factors. These include dog age, weight, sex, spay/neuter status, and physical activity level, as these factors (apart from spay/neuter status) have been differentially shown to impact relaxation times in human IVDs [8, 51, 52, 53, 54]. Breed and genetics are also a potential source of variation [15] and further studies that include genetic sequencing are needed to determine how genetics influence disc composition and whether breed impacts T2 relaxation times in a predictable, measurable way. Although our sample size is too limited to disentangle these various factors, there is some evidence that age contributed to some of the variation in quantitative MRI values between the dogs, as has also been observed in humans [55]. In particular, while most of the dogs in our sample were elderly, two of the youngest dogs (Dogs #7 and #8) had the longest T2 relaxation times in the NP. Furthermore, in vivo studies of T2 relaxation times in younger cohorts of dogs have reported longer T2 relaxation times in healthy discs than we found in our study [36, 37]. Further investigation of changes in relaxation times with aging in dogs is warranted.

Although T2 relaxation times of healthy discs looked different between dogs, they were similar between disc levels when analyzed across the population as a whole. Studies in humans have previously shown decreasing relaxation times when proceeding inferiorly down the spine [56, 57], a trend that likely reflects the increasing compressive forces that act on the vertically oriented spines of humans due to gravity. With the horizontal positioning of dog spines, it is unsurprising that the disc relaxation times we measured did not fit a similar cranial‐to‐caudal trend. Imaging of cervical and thoracic spinal segments would be necessary to determine whether any discs in the dog spine show significant relaxation time differences related to their positions within the spine. When looking at degenerated discs across disc levels, we found that the L7‐S1 disc level had a higher frequency of degeneration than the other disc levels. Positional imaging studies have revealed that L7‐S1 is the most mobile segment of the dog spine [58] and therefore is more likely to undergo degenerative changes [59]; however, our relaxation time results show that healthy L7‐S1 discs are not compositionally distinct from other disc levels. The similarity of relaxation times across disc levels suggests the potential ability to normalize T2 relaxation times on a per‐dog basis using a dog's other discs to control for variation between individuals.

Our study had several limitations. First, while our study population was sufficient to identify trends across dogs, disc levels, and disc health measures, it was still limited in overall sample size and in terms of representation across age, weight, breed, and spay/neuter status. Further studies with a larger population are needed to disentangle the contributions made by each patient‐related factor in determining an individual dog's IVD relaxation times. Second, our study was ex vivo and cross‐sectional. To expand on this work, in vivo longitudinal studies are necessary to evaluate the progressive degeneration of the intervertebral discs, to determine whether transient changes such as diurnal variation may occur, to assess the repeatability of measurements between scans, and to eliminate any post‐mortem effects that may have impacted relaxation times. Third, our analysis had technical limitations that may have contributed to variance in the data, such as heterogeneity of signal in the disc ROIs, partial volume averaging in axial imaging of thin discs, increased B_0_‐ and/or B_1_‐field inhomogeneity at the T11‐T12 and L7‐S1 disc levels, and the small size of tissue samples that were available for the biochemical assays. Quantitative MRI measurements that consider the heterogeneity of the signal in the disc and use sagittal acquisitions can be considered in the future [60]. Lastly, while our findings identified important changes in relaxation times, caution should be taken when interpreting absolute relaxation time values. Because the specimens were imaged ex vivo at room temperature, it can be expected that the relaxation times were shorter than they would be in vivo [61]. It is also important to note that absolute relaxation times are dependent on field strength, pulse sequence parameters, fitting methods, and other factors [62, 63]. In our study, these factors were all held constant so that our comparisons were not confounded by these differences, but direct comparison to other studies needs to consider these potential differences, in addition to differences in the sample population.

In conclusion, quantitative T2, T2*, T1ρ, adiabatic T1ρ, and adiabatic T2ρ relaxation time mapping techniques are similarly related to radiological and histological measures of disc health and water and GAG content in a general dog population. We determined that baseline relaxation times differ between dogs, and that clinically available T2 mapping can provide correlative information about disc health in dogs with disc degeneration after accounting for these differing baselines. These findings provide an important foundation for future research into the applications of quantitative imaging in dog spines and the translatability of client‐owned dogs as a clinical model for IVD degeneration. Our work supports that quantitative MRI may be a useful modality to quantify subtle and early changes in disc health in longitudinal and treatment efficacy studies, with the potential to enhance the diagnostic capabilities surrounding IVD degeneration in both human and veterinary medicine.

## Conflicts of Interest

The authors declare no conflicts of interest.

## Supporting information


**Data S1:** Spreadsheet of all of the quantitative MRI and disc health measurements used in the data analyses.

**Figure S1:** Quantitative MRI maps and H&E‐ and AB/PSR‐stained histological sections for each of the 10 discs for a second representative dog (Dog #6).
**Figure S2:** Quantitative MRI maps and H&E‐ and AB/PSR‐stained histological sections for each of the 10 discs for a third representative dog (Dog #13).

## Data Availability

A spreadsheet of all of the quantitative MRI and disc health measurements used in the data analyses is included as Supporting Information. The raw MRI and histology images will also be made available upon request to the corresponding author.
