# Supplementary material for: Relationship Between Quantitative MRI and Radiological, Histological, and Biochemical Measures of Intervertebral Disc Health in Client‐Owned, Nonchondrodystrophic‐Breed Dogs
Source: JOR Spine. 2025 Aug 13;8(3):e70105. doi: 10.1002/jsp2.70105 (PMC12350182; doi:10.1002/jsp2.70105)

Figure S1. Quantitative MRI maps and H&E- and AB/PSR-stained histological sections for each of the 10 discs for a second representative dog (Dog #6). This dog had a range of disc health across levels (Pfirrmann grade 2: T11-T12, T12-T13, T13-L1, L1-L2; Pfirrmann grade 3: L4-L5, L5-L6, L6-L7; and Pfirrmann grade 4: L2-L3, L3-L4, L7-S1). The findings are consistent to those detailed for the case in Figure 6.

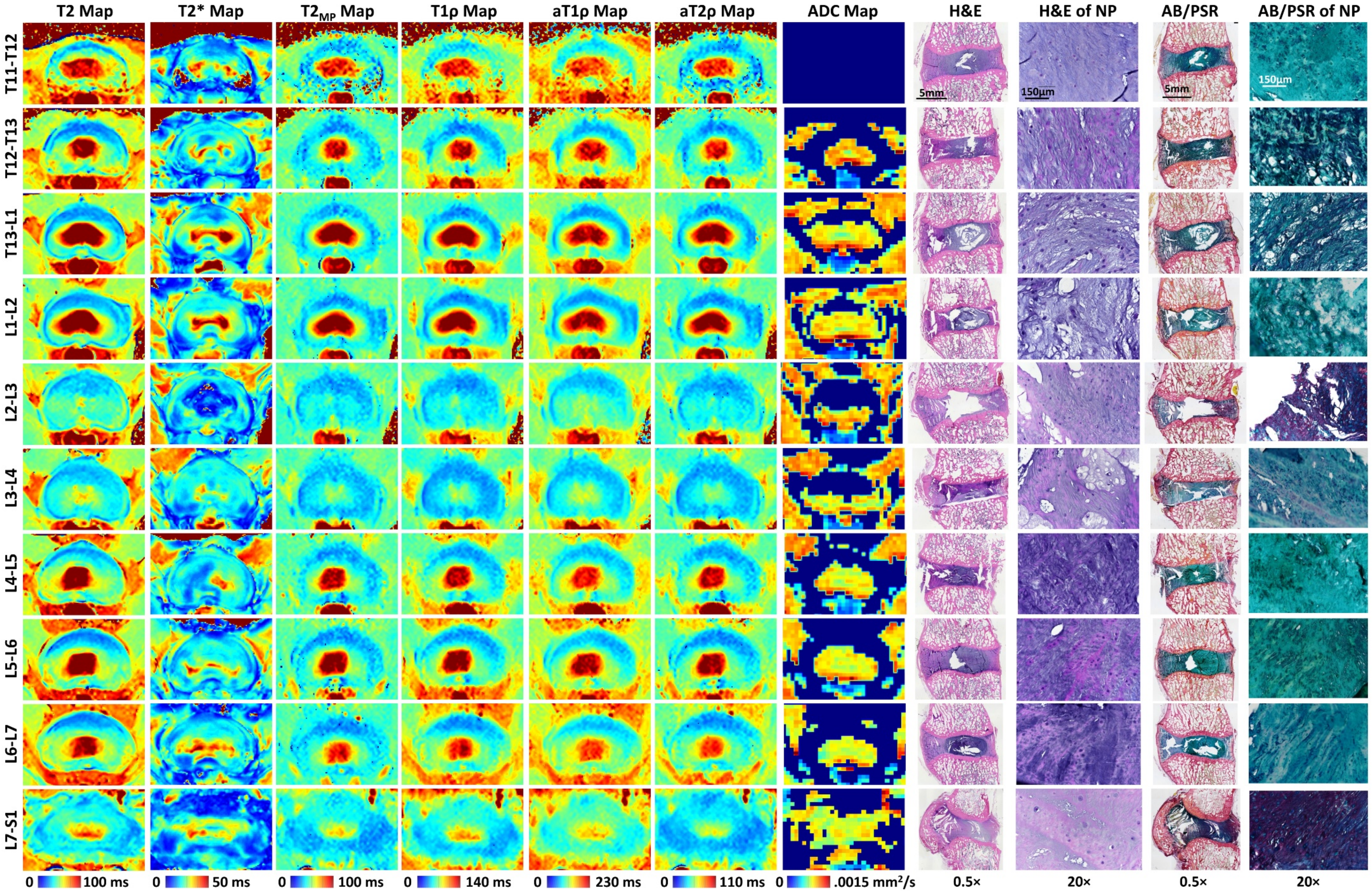

Figure S2. Quantitative MRI maps and H&E- and AB/PSR-stained histological sections for each of the 10 discs for a third representative dog (Dog #13). This dog had a range of disc health across levels (Pfirrmann grade 2: T11-T12, T12-T13, T13-L1, L2-L3, L3-L4, L4-L5, L5-L6, L6-L7; Pfirrmann grade 3: L1-L2, L7-S1). The findings are consistent to those detailed for the case in Figure 6.

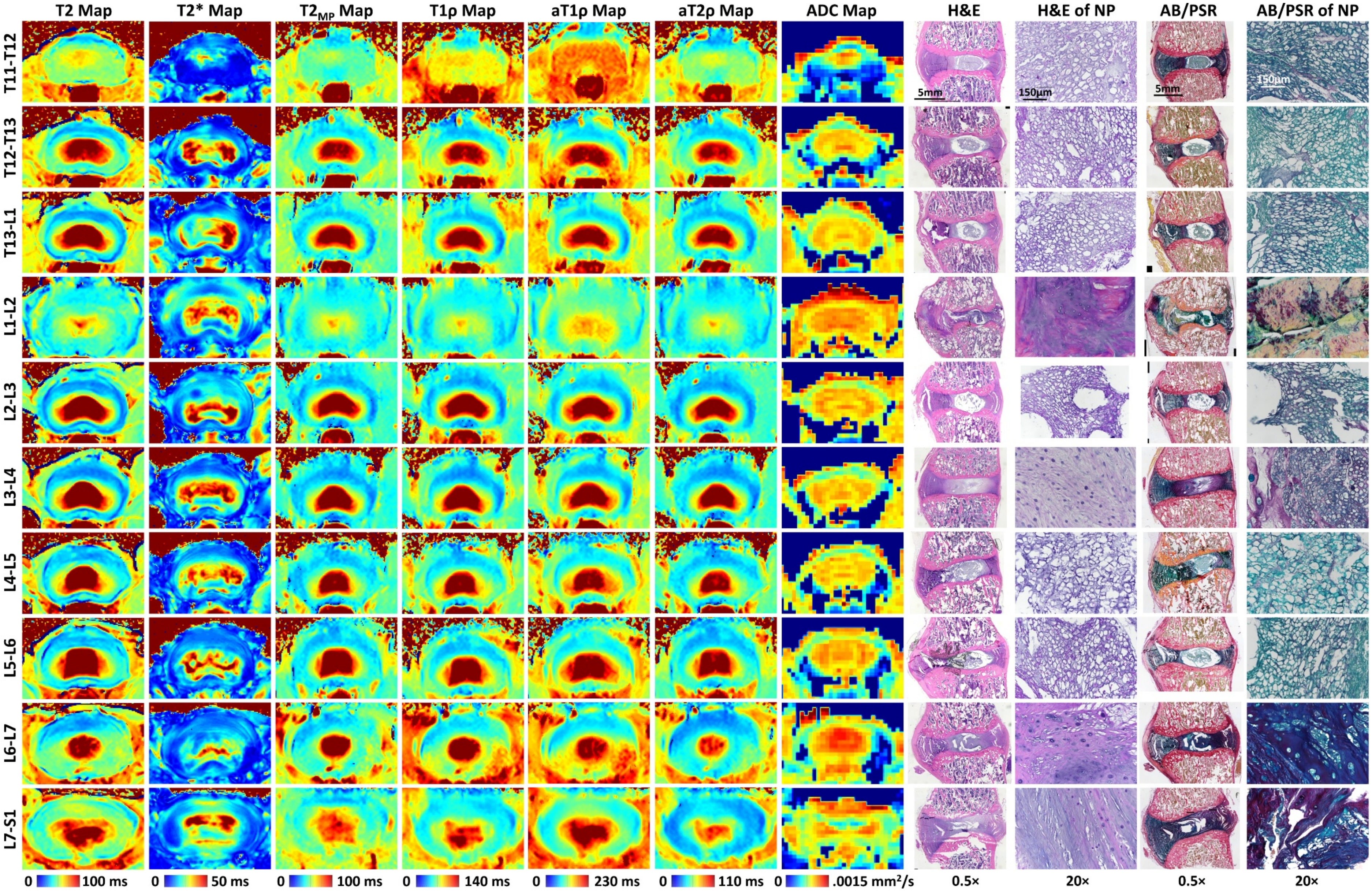

Supplement: Supplementary file 2 — Figure S1: Quantitative MRI maps and H&E‐ and AB/PSR‐stained histological sections for each of the 10 discs for a second representative dog (Dog #6). Figure S2: Quantitative MRI maps and H&E‐ and AB/PSR‐stained histological sections for each of the 10 discs for a third representative dog (Dog #13). [file JSP2-8-e70105-s002.pdf]
